# Sublethal Effects of the Insect Growth Regulator Novaluron on the Midgut Integrity and Survival of Adult Honey Bee *Apis mellifera* Workers

**DOI:** 10.1021/acsenvironau.5c00202

**Published:** 2026-02-26

**Authors:** Mateus Soares de Oliveira, João Victor de Oliveira Motta, Davy Soares Gomes, Giovanna dos Santos Pereira, Gabriel Martins Pantoja, Laryssa Lemos da Silva, João Paulo Pimentel de Oliveira Cruz, José Eduardo Serrão

**Affiliations:** † Department of General Biology, 28120 Federal University of Viçosa, Viçosa, Minas Gerais 36570-900, Brazil; ‡ Department of Entomology, Federal University of Viçosa, Viçosa, Minas Gerais 36570-900, Brazil

**Keywords:** chitin, benzoylurea, pesticide, histopathology, risk assessment

## Abstract

The insect growth regulator novaluron is a benzoylurea compound that disrupts the polymerization of chitin filaments. It is commonly used to control agricultural pests, particularly during their immature stages, and is generally considered nontoxic to adult insects. However, there is a lack of studies addressing the potential side effects of this insecticide on nontarget organisms, such as pollinating bees. In honey bees, the midgut is the primary organ responsible for digestion and nutrient absorption, where ingested food is surrounded by the peritrophic matrix, a structure composed of chitin microfibrils, glycosaminoglycans, and glycoproteins synthesized by digestive cells along the midgut. This study investigated whether chronic oral exposure to novaluron affects adult workers of the honey bee *Apis mellifera*. Specifically, we assessed the effects of the insecticide on the composition and permeability of the peritrophic matrix, the histopathology of the midgut, and worker mortality. Bees exposed chronically to a sublethal concentration of novaluron for 10 days showed reduced chitin levels in the peritrophic matrix, which appeared disorganized and diffuse, along with increased permeability of this barrier. Furthermore, exposed bees exhibited histopathological alterations in the midgut epithelium and elevated mortality rates. These findings indicate that, in the context of chronic oral exposure, commercial formulation of the insecticide novaluron, although classified as an insect growth regulator, is toxic to adult *A. mellifera* workers at the tissue level.

## Graphical abstract

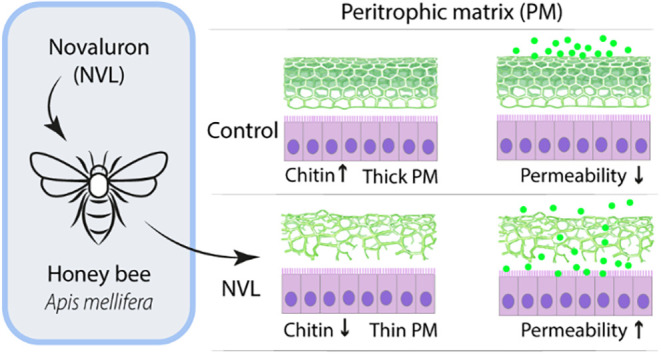

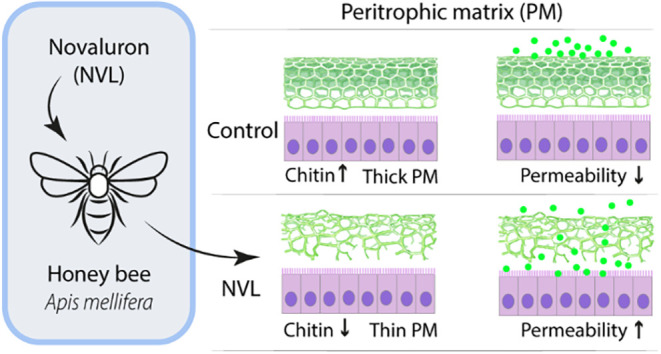

## 1 Introduction

Novaluron is an insect growth regulator (IGR) belonging to the benzoylurea group. It acts by disrupting the transport of specific proteins essential for chitin polymerization.^1^ Its use is recommended for controlling insect pests during their immature stages, including species of Lepidoptera, Coleoptera, Hemiptera, and Diptera,^1^ in a variety of crops such as apples, potatoes, pumpkins, zucchini, coffee, sugar cane, corn, soybeans, and others. Novaluron is considered a valuable alternative to organophosphate insecticides by both the U.S. Environmental Protection Agency (EPA) and the Canadian Pest Management Regulatory Agency (PMRA), primarily due to its low toxicity to nontarget insect species.^1^

The toxic effects of novaluron on pollinators have been primarily evaluated in juvenile stages,^2–5^ with no significant mortality reported in adult bees.^4,6^ Although data on the histotoxicity of novaluron in adult bees are lacking, the effects of pesticides on vital organs, particularly the midgut, have been widely documented.^7–15^

The midgut of *Apis mellifera* consists of a simple epithelium with a well-developed apical brush border. It contains columnar digestive cells responsible for enzyme secretion and nutrient absorption; endocrine cells with regulatory functions; and regenerative cells, organized in clusters at the basal region of the epithelium, which play a crucial role in replacing other cell types within the organ.^16,17^

The midgut lumen is lined by concentric, semipermeable, acellular layers forming a structure known as the peritrophic matrix, which divides the lumen into two distinct functional compartments: the endoperitrophic space, where the food bolus is located, and the ectoperitrophic space, situated between the matrix and the midgut epithelium. In the ectoperitrophic space, retrograde flow of fluids and digestive enzymes occurs, along with the reabsorption and recycling of these enzymes.^18,19^

The peritrophic matrix is composed of chitin microfibrils, glycosaminoglycans, glycoproteins, and various proteins, all synthesized by digestive cells along the midgut.^20–22^ This matrix plays a key role in optimizing the digestive process,^23^ protecting the epithelium from mechanical damage caused by the food bolus,^20^ and preventing infections by microorganisms,^18,24–27^ as well as exposure to toxins and other harmful substances.^28^

Chitin is a linear polysaccharide composed of *N*-acetylglucosamine units, synthesized from trehalose through a series of enzymatic reactions. It accounts for approximately 13% (w/w) of the peritrophic matrix.^22^ Chitin chains are tightly polymerized to form nanofibers,^29^ which subsequently associate into microfibrils. These microfibrils are typically organized in parallel arrays of 10 or more, forming robust bundles.^30–32^ This microfibrillar network serves as a scaffold onto which proteins containing chitin-binding domains attach, cross-linking with the microfibrils to establish the structural framework of the peritrophic matrix.^33,34^

Because it is composed of chitin microfibrils that are continuously synthesized in the midgut, the peritrophic matrix may serve as a secondary target for insect growth regulators. This has been reported in larvae of *Tribolium castaneum* (Coleoptera) and *Aedes aegypti* (Diptera), which exhibited altered peritrophic matrix permeability following exposure to novaluron.^35^

*Apis mellifera* is considered the most effective pollinator for crops worldwide due to its broad geographic distribution^36,37^ and its preference for nectar- and pollen-rich plants.^38–40^ As a result, it contributes directly to the increased productivity and profitability of numerous crops.^37,40–42^ However, global declines in bee populations have been reported,^43^ with multiple contributing factors, including pathogens and parasites,^44^ climate change,^45^ poor nutrition,^46^ excessive pesticide use,^47^ and synergistic interactions among these stressors.^48,49^

Pesticides have been identified as major stressors for bees, significantly contributing to the spread of pathogens and parasites by compromising bee health and weakening the immune system.^50,51^ Bees may be exposed to pesticides directly, through contact with airborne particles, or indirectly, by ingesting contaminated pollen and nectar.^52,53^ The effects of pesticide poisoning in colonies may manifest as mass die-offs^54^ or the sudden disappearance of bees from the hive, a phenomenon known as Colony Collapse Disorder.^55^

This study evaluated the side effects of chronic oral exposure to a novaluron-based insecticide on adult *A. mellifera* workers. Specifically, we investigated changes in the composition and permeability of the peritrophic matrix, midgut histopathology, and worker mortality.

## 2 Materials and Methods

### 2.1 Insects

To obtain newly emerged bees, three frames containing *A. mellifera* pupae were collected from three different colonies at the Central Apiary of the Federal University of Viçosa (UFV), Viçosa, Minas Gerais, Brazil (20°45′N; 42°52′E). The frames were maintained at 34 °C and 70% relative humidity for 24 h until adult emergence.^56^ Newly emerged *A. mellifera* workers were then transferred to 250 mL plastic jars, with 10 bees per jar, and maintained at 32 °C and 70% relative humidity.^56^ The bees were provided with *ad libitum* access to water, honey, and pollen for 72 h prior to being subjected to toxicological tests involving chronic oral exposure to the novaluron-based insecticide.

### 2.2 Insecticide Exposure

To evaluate whether environmental residue levels of novaluron in pollen and nectar have cytotoxic and physiological effects on bees, the estimated environmental concentration (EEC) was calculated using the Bee Residue Exposure (BeeREX) model, based on the application of the commercial insecticide formulation.^57^ The calculation was performed using the maximum recommended field concentration (300 mL ha^–1^) of the novaluron-based insecticide Rimon Supra (100 g L^–1^ active ingredient, 1016 g L^–1^ inert ingredients; ADAMA BRASIL S/A, Londrina, PR, Brazil), used to control the coffee leaf miner *Leucoptera coffeella* (Lepidoptera: Lyonetiidae) in coffee crops. The EEC estimated for coffee crops was used as a proxy to determine the concentration potentially present in pollen and nectar, resulting in a value of 2.94 ng a.i. mg^–1^. This concentration was used for the chronic oral exposure tests. Newly emerged *A. mellifera* workers (*n* = 40) were fed on 50% sucrose solution containing the estimated novaluron concentration, while control bees (*n* = 40) received only a 50% sucrose solution. The food was provided in 1.5 mL microtubes attached to 250 mL plastic jars containing 10 bees each,^56^ for a period of 10 days. The experiment was conducted in triplicate. The final sample size was 30 bees in the control group and 30 in the treated group. All bees dissected for histological, histochemical, and fluorescence analyses originated from three different colonies and were randomly collected from 250 mL plastic jars in which they were contained during the 10 days of chronic oral exposure to the insecticide or control. This allowed for colony-level replication and reduced genetic and physiological bias, increasing the biological representativeness and statistical robustness of the ecotoxicological results.

### 2.3 Survival Analysis

40 newly emerged bees from three different colonies were acclimatized for 72 h as described in Section 2.1. For the survival assay, bees were randomly assigned to control or novaluron treatments. In each experimental run, 40 bees per group were distributed into four 250 mL plastic jars, with 10 bees per jar, which were considered the experimental replicates. This procedure was repeated three independent times. Therefore, survival analysis was performed on a total of 240 bees, comprising 120 control individuals and 120 novaluron-exposed individuals. Newly emerged *A. mellifera* workers in the treatment group were fed a 50% sucrose solution containing the estimated environmental concentration (EEC) of novaluron-based insecticide Rimon Supra as calculated by the BeeREX model, while control bees received only 50% sucrose solution. Mortality was recorded every 24 h over the during 10 days of chronic exposure. Dead bees were removed and discarded promptly.

### 2.4 Histopathology and Histochemistry

Ten worker bees were collected and dissected after 10 days of oral exposure to novaluron-based insecticide Rimon Supra and from the control group. These bees, originating from three different colonies, were randomly collected from the 250 mL jars in which they were stored. The bees were cryo-anesthetized at −5 °C for 5 min, then dissected in 125 mM NaCl solution. The midguts were carefully removed and transferred to Zamboni’s fixative solution^58^ for 12 h. Samples were dehydrated through a graded ethanol series (70, 80, 90, and 95%) and embedded in Leica historesin. Semithin sections, 3 μm thick, were cut using glass knife on a rotary microtome (Leica RM2255), then stained with hematoxylin and eosin or submitted to the periodic acid-Schiff (PAS) technique for glycoconjugate detection.^59^ Sections were analyzed and photographed using an Olympus BX53 microscope.

### 2.5 Histochemical Quantification

For each analysis, one image was taken for each section analyzed, using the Olympus BX53 photomicroscope. Ten histological sections of the midgut from each of 10 control bees and 10 treated bees were evaluated.^60^ Images of the midgut sections submitted to the PAS test were analyzed for the intensity of the histochemical reaction in the peritrophic matrix using pixel intensity measurements with ImageJ/FIJI software. For analysis of the peritrophic matrix area, the same number of images per section, sections per intestine, and bees were maintained. The peritrophic matrix area was quantified using the “threshold” tool, which segments the image into distinct groups of pixels, commonly referred to as “foreground” and “background.” This segmentation facilitates the isolation of specific regions of interest within the image, enabling more precise analysis and extraction of relevant data. The measurements were averaged at the individual level prior to statistical analysis, and that each bee was treated as the experimental unit. This approach avoids pseudoreplication and ensures statistical robustness.

### 2.6 Chitin Quantification

To evaluate the effect of the novaluron-based insecticide Rimon Supra on chitin synthesis in adult *A. mellifera* workers, 10 treated bees and 10 control bees were obtained from an independent experiment. Their midguts were dissected, fixed in Zamboni’s solution,^58^ and processed for histological analysis as described above. Longitudinal sections, 5 μm thick, were incubated for 1 h with wheat germ agglutinin conjugated to fluorescein isothiocyanate (WGA-FITC; Sigma-Aldrich) in the presence of 0.2 M *N*-acetylglucosamine for chitin detection.^61^ Following incubation, sections were washed for 1 h in 0.1 M sodium phosphate buffer (pH 7.2), then washed again in 0.1 M sodium phosphate-buffered saline (PBS, pH 7.6). Nuclei were counterstained with 4′,6-diamidino-2-phenylindole (DAPI) at 0.2 μg mL^–1^ for 1 h, followed by a final wash in PBS. Samples were mounted and analyzed using an Olympus BX60 epifluorescence microscope. Chitin quantification was performed by measuring fluorescence intensity of 10 images per intestine obtained randomly from 10 different histological sections using ImageJ/FIJI software, following the protocol described by Oliveira et al.^60^ The measurements were averaged at the individual level prior to statistical analysis, and that each bee was treated as the experimental unit. This approach avoids pseudoreplication.

### 2.7 Qualitative Analysis of the Peritrophic Matrix Permeability

To assess the permeability of the peritrophic matrix, 30 adult *A. mellifera* workers from both the control and treatment groups were collected and transferred to 250 mL plastic cages in groups of 10 individuals.^62^ They were fed either a 50% sucrose solution containing the novaluron-based insecticide Rimon Supra (2.94 ng a.i. mg^–1^) or a 50% sucrose solution as control, for 10 days. Following exposure, bees were starved for 3 h, then individually isolated in 0.2 mL microtubes and fed 5 μL of 50% sucrose solution containing dextran-FITC conjugate (molecular weight 70 kDa, at 1 mg mL^–1^) using an automatic pipet.^56^ This dextran-FITC conjugate is known not to cross the peritrophic matrix of *A. mellifera*.^60^ One hour after ingestion, bees were dissected, and longitudinal sections (5 μm thick) were prepared as described above. Nuclei were stained with DAPI, and the samples were analyzed using an Olympus BX60 epifluorescence microscope.

### 2.8 Statistical Analysis

Pixel intensity data from the quantification of glycoconjugates in the PAS histochemical test, the area of the peritrophic matrix, and the fluorescence intensity of WGA-FITC were first assessed for normality using the Shapiro-Wilk test and for homogeneity of variances using Levene’s test. The t’Student test was applied to analyze pixel intensity data for glycoconjugate quantification and WGA-FITC fluorescence intensity. For the peritrophic matrix area, data were analyzed using the nonparametric Mann–Whitney U test. Mortality data were analyzed using survival analysis with the Kaplan–Meier estimator (log-rank test). Cox regression analysis was performed to estimate the survival probability of the control group relative to the treated group. All analyses were performed using bees originating from three different colonies, with individuals randomly distributed among experimental units, ensuring biological replication and minimizing potential colony-specific bias. All statistical analyses were conducted using Jamovi version 2.3.18, with a significance level set at 5%.

## 3 Results

### 3.1 Survival

The survival of newly emerged *A. mellifera* workers orally exposed to the insecticide for 10 days was significantly reduced, decreasing from 87.5% in the control group to 49.2% in the novaluron-treated group (log-rank test, χ^2^ = 38.2; d.f. = 1; *p* < 0.001; Figure 1). Cox regression analysis revealed that bees in the treated group had a 5.01-fold higher risk of mortality compared to controls (95% C.I. = 2.84 – 8.85, *p* < 0.01).

**Figure 1:**
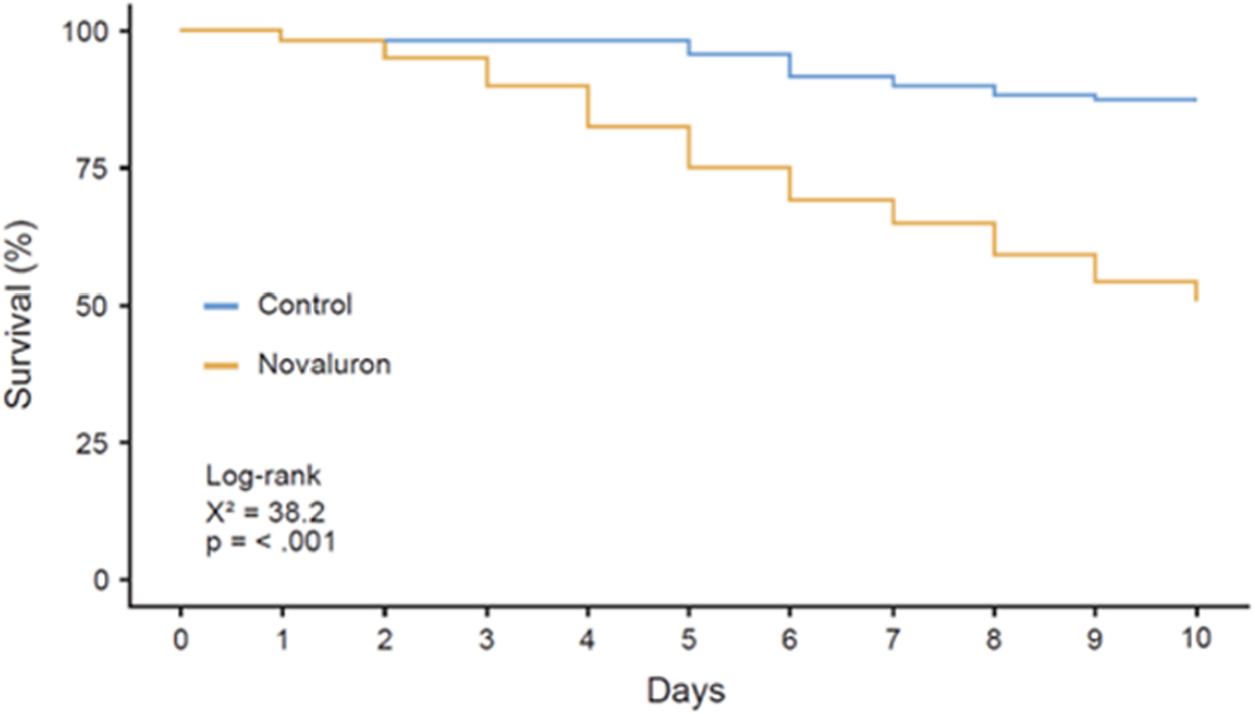
Kaplan–Meier survival analysis. Mortality of newly emerged *Apis mellifera* bees orally exposed to 50% sucrose solution and novaluron (2.94 ng a.i. mg^–1^) for 10 days (log-rank = 38.2, *p* < 0.001).

### 3.2 Histopathology

*Apis mellifera* workers from the control group, fed an insecticide-free sucrose solution for 10 days, exhibited a midgut epithelium composed of columnar digestive cells with nuclei containing decondensed chromatin and prominent nucleoli, a well-developed apical brush border, and nests of regenerative cells located in the basal region of the epithelium (Figure 2A,B). Multiple layers of the peritrophic matrix were observed lining the lumen (Figure 2A,B).

**Figure 2:**
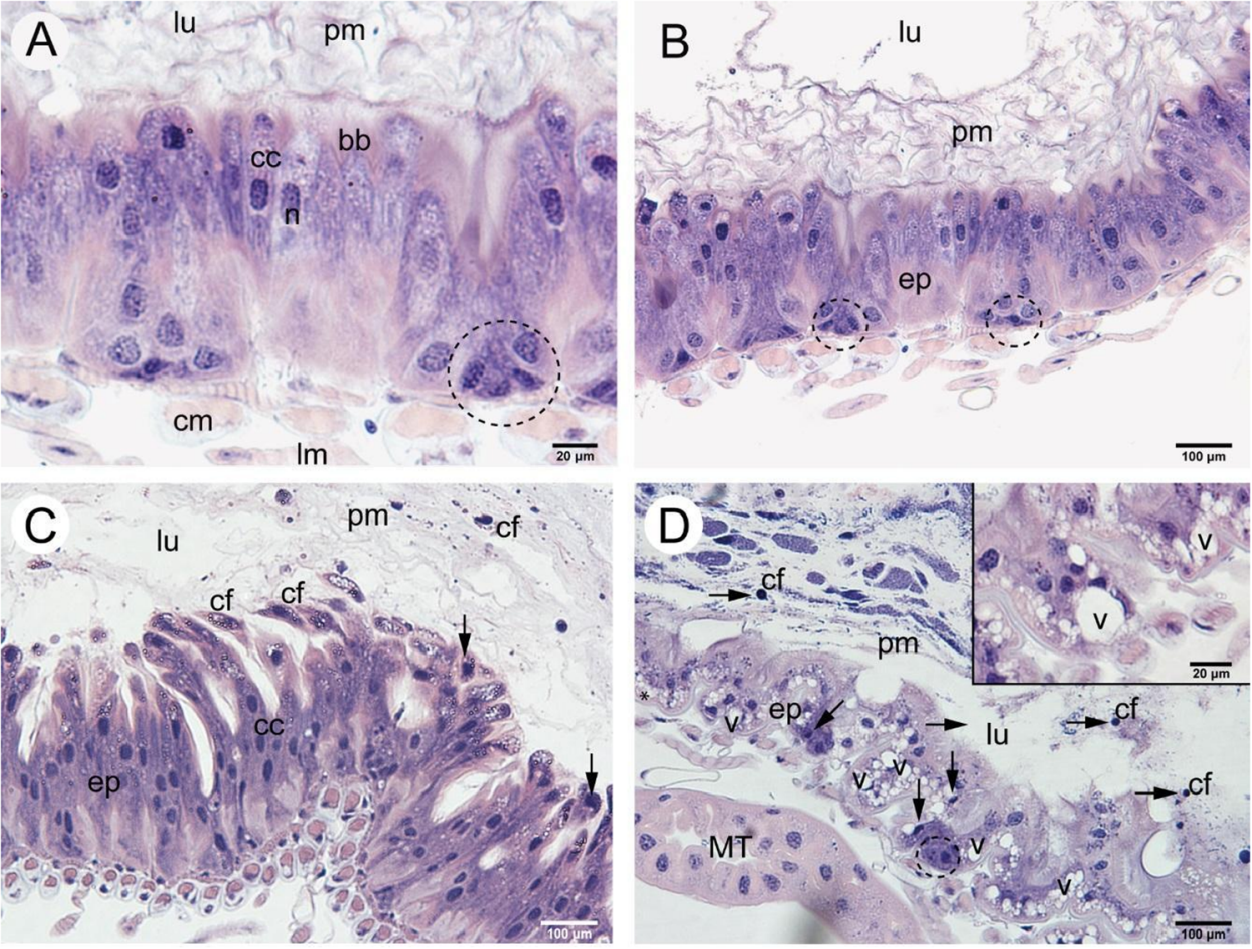
Light micrographs of the midgut of *Apis mellifera* workers dissected after 10 days of chronic exposure. [A, B]: Control bees, showing healthy epithelium (ep) with columnar digestive cells (cc), nuclei (n) with decondensed chromatin, brush border (bb), nests of regenerative cells (dotted circles) and lumen (lu) with organized layers of peritrophic matrix (pm). [C, D]: Treatment. Highly damaged epithelium (ep), showing many vacuoles (v), nuclear pyknosis (arrows), diffuse and disorganized peritrophic matrix (pm) and cell fragments (cf) with pyknotic nuclei, escaping into the lumen (lu). cm = circular musculature. lm = longitudinal musculature. MT = Malpighian tubules. Hematoxylin and eosin.

Bees exposed to the novaluron-based insecticide Rimon Supra for 10 days displayed histopathological alterations in the midgut epithelium. Some intestines showed disorganization of epithelial architecture, with digestive cells exhibiting cytoplasm rich in vacuoles being released into the lumen (Figure 2C), nuclear pyknosis (Figure 2C), and cell fragments present within the lumen (Figure 2D). More advanced histopathological signs were observed in some samples, including disruption of cell-to-cell contact resulting in dilated intercellular spaces and a reduction in the number of regenerative cell nests (Figure 2D). Additionally, the peritrophic matrix in treated bees appeared diffuse and disorganized (Figure 2C,D).

### 3.3 Histochemistry

In bees treated with the novaluron-based insecticide Rimon Supra, the PAS histochemical test for glycoconjugates revealed a significantly lower staining intensity in the peritrophic matrix compared to control bees (Student’s *t* test = 2.38, d.f. = 18, p = 0.02, 95% confidence level; Figure 3A–C). Additionally, the area of the peritrophic matrix within the midgut lumen was significantly reduced in insecticide-exposed bees relative to controls (Mann–Whitney *U* = 30512, *p* < 0.001; Figure 3D).

**Figure 3:**
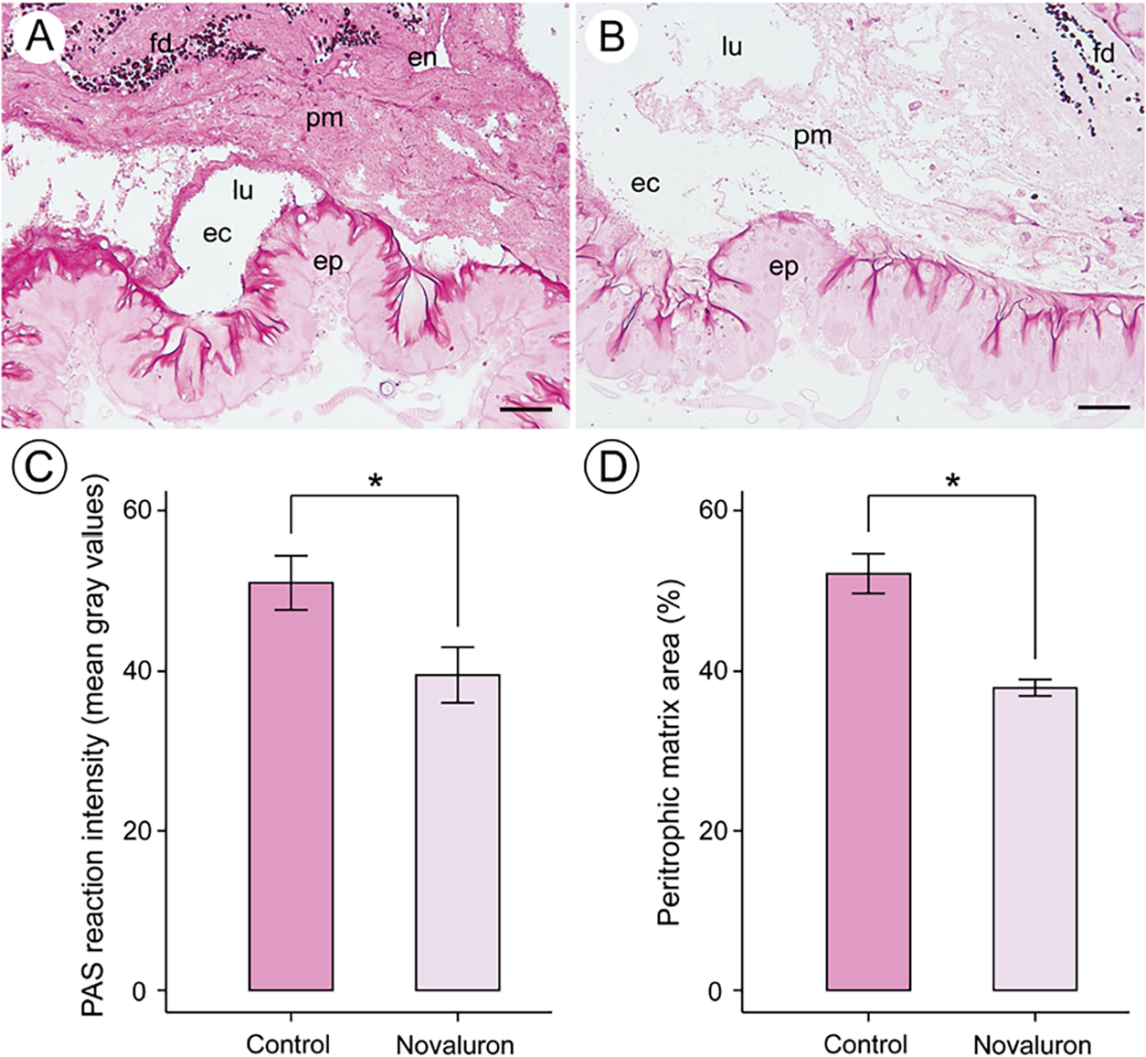
Peritrophic matrix of *Apis mellifera* submitted to PAS histochemical test and quantification. [A-B]: Light micrographs of the midgut of *A. mellifera*. [A]: Control showing lumen (lu) with peritrophic matrix (pm) delimiting the endoperitrophic (en) and ectoperitrophic (ec) spaces. [B]: Insecticide-treated bees showing less intense positive reactions for carbohydrates and glycoconjugates in the peritrophic matrix (pm) compared to the control group (A). [C]: Quantification of the histochemical reaction by pixel intensity. [D]: Quantification of the peritrophic matrix area in relation to the visible lumen in histological sections. ep = epithelium. fd = food. Scale bars 50 μm.

### 3.4 Chitin Quantification

WGA-FITC was used to specifically label *N*-acetylglucosamine, enabling a visual and semiquantitative assessment of chitin in the peritrophic matrix of bees orally exposed to the novaluron-based insecticide for 10 days, as well as in control bees. The fluorescence intensity in the peritrophic matrix of insecticide-treated bees was significantly lower than that of the controls (Student’s *t* test = 3.00, d.f. = 18, *p* = 0.008, 95% confidence level; Figure 4), indicating a reduction in chitin content.

**Figure 4:**
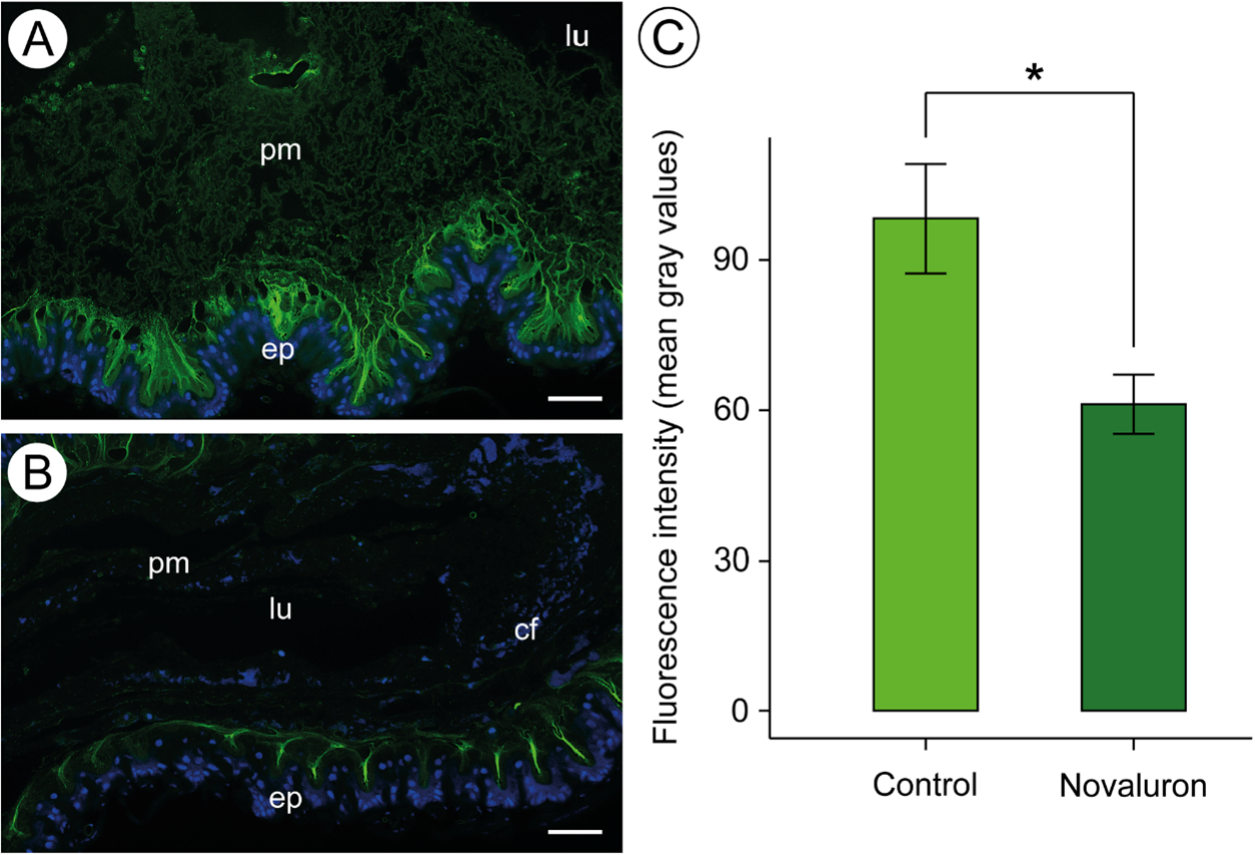
Fluorescence intensity and semiquantification of chitin in the peritrophic matrix of *Apis mellifera*. [A-B]: midgut subjected to wheat germ agglutinin (WGA) (green) to detect chitin and DAPI (blue) to mark nuclei. [A]: Control showing epithelium (ep) and lumen (lu) with fluorescence-reactive peritrophic matrix (pm). [B]: bee midgut treated with insecticide showing less intense positive reactions for chitin in the peritrophic matrix (pm) compared to the control group (A). [C]: Semiquantification of chitin by measuring pixel intensity. * Indicates significant difference by Student’s *t* test, *p* < 0.05. lu = lumen. cf = cell fragment. Scale bars = 50 μm.

### 3.5 Qualitative Analysis of the Peritrophic Matrix Permeability

In the control group, FITC-conjugated 70 kDa dextran molecules were confined to the endoperitrophic space, delimited by the peritrophic matrix (Figure 5A). In contrast, in the midguts of bees treated for 10 days with the growth regulator insecticide, FITC-dextran molecules were detected in the ectoperitrophic space, adjacent to the midgut epithelium surface (Figure 5B), indicating increased permeability of the peritrophic matrix to the molecule.

**Figure 5:**
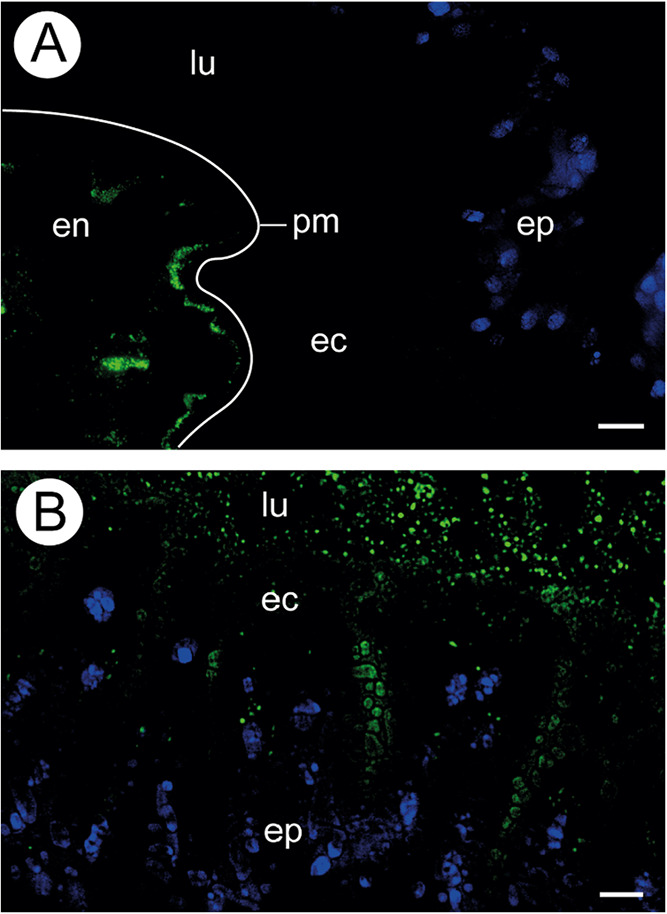
Sections of the midgut of *Apis mellifera* workers fed on FITC-dextran. FITC-dextran molecules of 70 kDa (green) are located in the endoperitrophic space (en) in the control group (A) and in the ectoperitrophic space (ec) in the treated group (B). Epithelium (ep) with nucleus DAPI-stained (blue). The white line shows the location of the peritrophic matrix. Scale bars = 20 μm.

## 4 Discussion

This study demonstrated that adult *A. mellifera* workers orally exposed to the field-relevant concentration of the novaluron insecticide formulation Rimon Supra for 10 days exhibit significant alterations in midgut integrity. These alterations include a reduction in glycoprotein and chitin content within the peritrophic matrix, histopathological damage, and increased mortality. These findings indicate that the commercial formulation of the insecticide novaluron, at a residual concentration of 2.94 ng a.i. mg^–1^, a growth inhibitor traditionally considered selective and primarily targeting immature insect stages, can exert significant adverse effects on nontarget adult insects such as bees.

The estimated environmental concentration (EEC) was calculated using the Bee Residual Exposure (BeeREX) model to assess whether the levels of environmental residues of novaluron used in this study (2.94 ng mg^–1^, equivalent to 2.94 ppm), estimated using BeeREX, is consistent with values measured empirically in the field. Fine et al.^5^ reported average residues of 3.38 ± 0.68 ng mg^–1^ in pollen from apple trees treated during flowering, indicating that the experimental scenario adopted is environmentally plausible and ecologically relevant.

The damage caused by chronic oral exposure to the field-relevant concentration of the insecticide Rimon Supra in the midgut of adult *A. mellifera* workers may have contributed to the reduced survival observed, with a 5-fold increase in mortality risk compared to control bees. This elevated mortality is likely a consequence of continuous oral exposure impairing the production of the peritrophic matrix, leading to epithelial damage and jeopardizing the regenerative capacity. Although growth-regulating insecticides are generally considered selective and are not typically associated with high mortality in adult insects, it is important to note that most studies on adult survival involve individuals exposed during their juvenile stages.^77–81^ In this study, the observed intestinal alterations, including increased vacuolization, nuclear pyknosis, and cell fragmentation, indicate that peritrophic matrix disruption and epithelial degeneration compromise essential digestive functions.

After 10 days of exposure to the field relevant concentration of the novaluron-based insecticide Rimon Supra, bees exhibited histopathological changes in the midgut epithelium, including digestive cells with intensely vacuolated cytoplasm, cell fragments being released into the gut lumen, nuclear pyknosis, loss of cohesion between cells resulting in enlarged intercellular spaces, and a reduction in the abundance of regenerative cell nests. Similar midgut histopathological damage has been reported in bees exposed to teflubenzuron,^60^ an insecticide with a mode of action analogous to novaluron.

The increase in cytoplasmic vacuoles and the presence of cell fragments in the midgut lumen of treated bees suggest the activation of a detoxification process involving the removal of toxic metabolites derived from insecticide metabolism into the midgut lumen.^63^ The occurrence of nuclear pyknosis is indicative of cell death by apoptosis.^64^ The observed decrease in regenerative cell nests may be linked to the differentiation of these cells into digestive cells^65^ as a compensatory response to increased apoptosis and cell loss in the lumen. Bee midgut cells possess molecular mechanisms capable of neutralizing xenobiotic compounds, including enzymes such as cytochrome P450 monooxygenases, glutathione S-transferases, and carboxylesterases/cholinesterases, which play fundamental roles in pesticide metabolism.^66–68^ However, under conditions of high pesticide levels or prolonged exposure, these detoxification enzyme systems can become overwhelmed, leading to a decline in their activities.^69^ The morphological alterations observed in the guts of pesticide-exposed bees appear to be correlated with the inability of digestive cells’ detoxification mechanisms to cope with insecticide overload.

The insecticide Rimon Supra is coformulated with a powerful solvent that can increase epithelial penetration and have other toxic effects of concern.^5^ Our results indicate a cytotoxic effect of the commercial formulation of the insecticide Rimon Supra. Therefore, the possibility of cytotoxic effects of the coformulant in Rimon Supra cannot be ruled out or ignored.

The reduced intensity of the PAS reaction observed in the peritrophic matrix of bees treated with the insecticide suggests a decrease in glycoconjugates within this structure. The peritrophic matrix is a critical component of the insect midgut, primarily composed of chitin and glycoproteins arranged in successive concentric layers. It serves to protect the midgut epithelium against pathogens, mechanical abrasion, and toxic substances, while also regulating digestion, nutrient absorption, and maintaining organ homeostasis by acting as both a physical and biochemical barrier between the lumen and the epithelium.^26,28,32,70^ The observed reduction in glycoconjugate incorporation likely explains the peritrophic matrix’s diffuse aspect and the decreased area it occupies within the lumen, reflecting diminished matrix production. These findings indicate that chronic exposure to field-relevant concentrations of the novaluron formulation Rimon Supra compromises the integrity and functionality of the peritrophic matrix, rendering the midgut epithelium more susceptible to damage, infection, and impairments in digestion and nutrient absorption, which can directly impact bee health and survival.

The decrease in glycoconjugates in the peritrophic matrix of bees exposed to the insecticide may also be linked to energy mobilization for detoxification processes.^61,71,72^ Therefore, the reduced PAS reaction observed in the peritrophic matrix of bees treated with the growth inhibitor does not necessarily reflect a direct effect on glycoconjugates such as chitin within the peritrophic matrix. To more precisely assess the impact of Rimon Supra on the composition and structure of the peritrophic matrix, midguts from exposed bees were analyzed using WGA-FITC staining, which has a high affinity for chitin. This analysis revealed a significant decrease in fluorescence intensity in the peritrophic matrix of treated bees. The reduced fluorescence suggests a decline in chitin microfibrils, indicating that novaluron affects the synthesis of this polysaccharide by midgut digestive cells by inhibiting the polymerization of *N*-acetylglucosamine monomers. Novaluron, like other benzoylurea insecticides, targets the sulfonylurea receptor (SUR) located on the membrane of intracellular vesicles involved in chitin filament polymerization and exocytosis.^73,74^ Although its primary target is juvenile insect stages, by preventing chitin synthesis essential for new cuticle formation during growth, continuous ingestion by adult bees can interfere with the renewal of the peritrophic matrix, a structure constantly synthesized by midgut cells throughout the insect’s life.^21,32^

The effect of Rimon Supra on the formation of the peritrophic matrix in adult *A. mellifera* workers led to evidence of increased permeability of this barrier, as demonstrated by the crossing of 70 kDa FITC-dextran molecules. An adequate concentration of chitin is essential to ensure the barrier function of the peritrophic matrix; thus, greater inhibition of chitin synthesis results in increased permeability to molecules and particles that, under normal conditions, would be retained within the endoperitrophic space.^35^ By compromising the structural integrity and barrier function of the peritrophic matrix, the novaluron-based insecticide may expose the intestinal epithelium to pathogens, toxins, and digestive byproducts, leading to physiological stress, digestive dysfunction, and histopathological damage. Increased permeability of the peritrophic matrix has been associated with a higher susceptibility to *Nosema ceranae* infection in *A. mellifera*,^27^ *Nosema pernyi* in *Antheraea pernyi* (Lepidoptera),^75^ and baculovirus infections in Lepidoptera larvae of *Trichoplusia ni* and *Pseudaletia unipuncta*.^76^ Although a quantitative or semiquantitative analysis of peritrophic matrix permeability was not feasible in this study, the permeability results are qualitative and based on multiple images of 30 bees in replicates, providing robust data that infer increased permeability. However, we reinforce the need for further studies that provide a quantitative perspective on the permeability of this barrier.

These findings underscore that compounds interfering with chitin synthesis, even at environmentally realistic concentrations, can negatively impact the health and survival of adult bees. In this study, only one sublethal concentration was tested. Although justified as field-relevant, this limits inference. Therefore, further studies should be conducted evaluating different environmentally relevant concentrations.

## 5 Conclusion

Given the ecological importance of bees as pollinators and the widespread use of insect growth regulators as alternatives to organophosphates, based on the assumption of their low toxicity to nontarget insects,^1^ our findings highlight the need to broaden investigations into the sublethal effects of these compounds. Ecotoxicological assessments of pesticides should go beyond mortality rates and include analyses of morphological and physiological alterations that may compromise the health, behavior, and long-term functionality of bee colonies.
